# Supplementary material for: Active screening significantly improves detection of chlamydia in women’s health clinics in Shaoxing, China
Source: PLoS One. 2025 May 7;20(5):e0321492. doi: 10.1371/journal.pone.0321492 (PMC12072664; doi:10.1371/journal.pone.0321492)
Supplement: Attachment — Submitted filename: Response to reviewers.docx [file pone.0321492.s004.docx]

2024/10/8

Response for manuscript PONE-D-24-27749 “Active screening significantly improves detection of chlamydia in women' s health clinics in Shaoxing, China”

**Dear reviewer**

Thank you very much for your comments and professional advice. These opinions help to improve academic rigor of our article. Based on your suggestion and request, we have made corrected modifications on the revised manuscript. Meanwhile, the manuscript had be reviewed and edited by language services of native English speakers. We hope that our work can be improved again. Furthermore, we would like to show the details as follows:

**Reviewer 1#**

-The authors have addressed an issue that continues to be topical - chlamydia trachomatis infection and its impact on reproductive health.

There are numerous typographical errors. The authors are advised to kindly address these.

Response: We feel sorry for our carelessness. We have carefully checked the grammar and spelling, and we also feel great thanks for your point out.

**Reviewer 2#**

I commend the authors for undertaking this study. In this research work, authors employed a hospital-based survey to compare the outcomes before and after the implementation of a chlamydia trachomatis screening protocol among women in Shaoxing Maternity and Child 38 Health Care Hospital.

-Extensive language editing is required. This has affected the write up in several ways communicating different things to a reader, eg unclear thoughts, loss of meaning and ambiguous conclusions and to mention a few. Authors should consider engaging a certified English language expert to help in the revision of the work.

Response: Thank you for this suggestion. The manuscript had be reviewed and edited by the native English speakers, we hope that the new version of the article has a better reading experience.

-Methodology

Who was involved in taking the samples per the screening protocol? This will be helpful to readers.

The statement in lines 131-132 ‘Chi-square test was used for qualitative data and t-test was used for quantitative data’ appears unclear to communicate the actual meaning to readers. Authors should kindly clarify what they really mean by this.

Response: Thank the reviewer very much for pointing these out to us! We have added the screening protocol about taking the samples and the statistical analysis in the methodology.

“This study included women aged 16 years and older who attended these outpatient clinics and were willing to undergo testing for C. trachomatis following an educational intervention by their attending physician. Individuals taking antibiotics or duplicate testing for C. trachomatis in the last month were excluded.”(line 79-83)

“Differences between T1 and T2 were assessed with the t-test (age) and the Chi-square test (ratio). ”(line 100-101)

-Results and discussion

Findings from this research work, indicate an increase in the number of people being screened for CT in reproductive health-related clinics as illustrated in T1 (7.235) and T2 (15,017). However, there is a marginal decline in the detection of positive rates of CT in the post-intervention phase, 5.2% (774/15005), compared to before the screening program, 6.8% (491/7230). An increase in the count data in T1 screening does not necessarily suggest an increase in rates. It can be inferred from the analysis that, screening for CT has increased, but the positive detection rates have not statistically increased after the introduction of the screening strategy protocol. Authors should relook at the analysis, interpret it in that light, and subsequently discuss their findings.

Response: Thank you for your insightful comment and kind suggestion. We have added the suggested content to the manuscript on discussion.

“These findings indicate that while CT screening has increased, the positive detection rates have not demonstrated a statistically significant increase after introducing the screening strategy protocol, which may be because doctors primarily detected C. trachomatis in symptomatic women and overlooked C. trachomatis detection in asymptomatic women during T1, while it is more likely to be detected in symptomatic women. Additionally, from a city-wide perspective, the significant increase in positive cases in our hospital will increase the incidence of C. trachomatis infection in the city, making the rate closer to the real situation.”(line 160-167)
